# Physiological effects of different recruitment maneuvers in a pig model of ARDS

**DOI:** 10.1186/s12871-020-01164-x

**Published:** 2020-10-21

**Authors:** Feiping Xia, Chun Pan, Lihui Wang, Ling Liu, Songqiao Liu, Fengmei Guo, Yi Yang, Yingzi Huang

**Affiliations:** Department of Critical Care Medicine, Zhongda Hospital, School of Medicine, Southeast University, No.87, Dingjiaqiao Road, Gulou District, Nanjing, 210009 Jiangsu China

**Keywords:** Electrical impedance tomography, Global inhomogeneity, Acute respiratory distress syndrome

## Abstract

**Background:**

In acute respiratory distress syndrome (ARDS), lung recruitment maneuvers can recruit collapsed alveoli in gravity-dependent lung regions, improving the homogeneity of ventilation distribution. This study used electrical impedance tomography to investigate the physiological effects of different recruitment maneuvers for alveolar recruitment in a pig model of ARDS.

**Methods:**

ARDS was induced in ten healthy male pigs with repeated bronchoalveolar lavage until the ratio of arterial partial pressure of oxygen (PaO_2_) of fraction of inspired oxygen (P/F) was < 100 mmHg and remained stable for 30 min (T_ARDS_). ARDS pigs underwent three sequential recruitment maneuvers, including sustained inflation, increments of positive end-expiratory pressure (PEEP), and pressure-controlled ventilation (PCV) applied in random order, with 30 mins at a PEEP of 5 cmH_2_O between maneuvers. Respiratory mechanics, hemodynamics, arterial blood gas, and electrical impedance tomography were recorded at baseline, T_ARDS_, and before and after each recruitment maneuver.

**Results:**

In all ten pigs, ARDS was successfully induced with a mean 2.8 ± 1.03 L bronchoalveolar lavages. PaO_2_, P/F, and compliance were significantly improved after recruitment with sustained inflation, increments of PEEP or PCV (all *p* < 0.05), and there were no significant differences between maneuvers. Global inhomogeneity index significantly decreased after recruitment with sustained inflation, increments of PEEP, or PCV. There were no significant differences in global inhomogeneity before or after recruitment with the different maneuvers. The decrease in global inhomogeneity index (ΔGI) was significantly greater after recruitment with increments of PEEP compared to sustained inflation (*p* = 0.023), but there was no significant difference in ΔGI between increments of PEEP and PCV or between sustained inflation and PCV.

**Conclusion:**

Sustained inflation, increments of PEEP, and PCV increased oxygenation, and regional and global compliance of the respiratory system, and decreased inhomogeneous gas distribution in ARDS pigs. Increments of PEEP significantly improved inhomogeneity of the lung compared to sustained inflation, while there was no difference between increments of PEEP and PCV or between sustained inflation and PCV.

## Background

Acute respiratory distress syndrome (ARDS) is a clinical syndrome characterized by a decrease in functional lung size [1]. The pathophysiology of ARDS includes diffuse alveolar collapse [2] and acute exudative lesions distributed in a gravitationally dependent gradient [3]. Although this disease was first defined almost 50 years ago, the hospital mortality rate for patients with severe ARDS remains high, estimated at 46% [4].

Lung recruitment maneuvers, including sustained inflation, increments of positive end-expiratory pressure (PEEP), and pressure-controlled ventilation (PCV), can improve oxygenation and increase respiratory system compliance in patients with ARDS. Recruitment maneuvers can recruit collapsed alveoli in gravity-dependent lung regions and improve the homogeneity of ventilation distribution, but may cause alveolar overdistention and lead to ventilator-associated lung injury in non-dependent regions [5]. A randomized controlled trial showed that sustained inflation and PCV improved the arterial partial pressure of oxygen (PaO_2_)/fraction of inspired oxygen (FiO_2_) (P/F) in 40 patients with ARDS, and the P/F was significantly increased after PCV compared to sustained inflation [6]. However, dynamic regional information on changes in lung ventilation after recruitment maneuvers has not been reported.

Recruitment and overdistention during lung recruitment have been evaluated by chest X-ray, computed tomography, and lung ultrasound. Electrical impedance tomography (EIT) is a non-invasive, radiation-free technique that can be used for bedside monitoring of lung tissue aeration during breathing. EIT allows semi-continuous, real-time measurement of changes in electrical resistivity within lung tissue and provides information on regional ventilation distributions [7, 8]. Domenighetti [9] reported that EIT can be used to measure impedance changes and assess regional ventilation distribution during tidal breathing. The EIT-based global inhomogeneity index has been developed as a tool to quantify tidal volume (Vt) distribution within the lung [10].

Previous research has focused on the effect of recruitment maneuvers on gas exchange and hemodynamics. Literature describing the influence of recruitment maneuvers on global inhomogeneity and regional ventilation distribution is scarce. This study used EIT to investigate the physiological effects of different recruitment maneuvers that achieve the same maximum pressure for alveolar recruitment in a porcine model of ARDS. Findings may inform clinical decision-making around recruitment maneuvers while minimizing the risk of barotrauma in individuals with ARDS.

## Methods

The protocol for this study was approved by the Science and Technological Committee and the Animal Use and Care Committee of the University School of Medicine, Nanjing, China. Domestic pigs (*Sus scrofa domesticus*) were purchased from a local farmer (Qinglongshan animal breeding farm, JiangShu, China). Animal experiments were performed in accordance with the Guidance for the Care and Use of Laboratory Animals [11].

### Animal preparation

Pigs were housed on straw in a cage and fed with a standard diet [12]. Prior to the study, the animals were fasted overnight. Ten healthy male pigs (body weight 50.3 ± 1.5 kg) were anesthetized with an intramuscular injection of ketamine hydrochloride (3 mg/kg), atropine (2 mg/kg) and fentanyl citrate (2 mg/kg) and an intravenous infusion of propofol (1–2 mg/kg·h), fentanyl citrate (0.5–1 μg/kg·h), midazolam (0.1 mg/kg·h), and atracurium (0.4 mg/kg·h) and placed in the supine position on a thermo-regulated operating table. During surgery, pigs received balanced electrolyte solution (5 ml/kg/h), pigs’ body temperature was maintained at 37.5 °C, and pigs’ mean arterial pressure (MAP) was maintained > 60 mmHg with rapid infusions of 0.9% saline (20 ml/kg), as needed.

Following anesthesia, tracheotomy was performed, and pigs were mechanically ventilated (Servo-i ventilator, Solna, Sweden) using volume-control mode at a Vt of 6 mL/kg, a respiratory rate of 30 breaths/min, FiO_2_ of 1.0, a inspiration-to-expiration time ratio (I:E) of 1:2, and PEEP of 5 cmH_2_O. Arterial blood samples were collected using a thermistor-tipped Pulse Contour Cardiac Output (PiCCO) catheter (Pulsion Medical System, Munich, Germany) inserted in the right femoral artery. Central venous pressure (CVP) and pulmonary arterial wedge pressure (PAWP) were measured using a Swan–Ganz catheter (Arrow International, Reading, PA, USA) inserted in the internal jugular vein. Cardiac output was measured with the Swan–Ganz catheter, and MAP was monitored with the PiCCO catheter.

### Experiment protocol

Baseline measurements (T_Baseline_) were made after pigs had stabilized for 30 min. Subsequently, a pig model of ARDS was established using bilateral lung lavage with isotonic saline (30 ml/kg; 38 °C) infused through a funnel. Negative pressure was applied to the proximal portion of an endotracheal tube to remove excessive fluid. Alveolar lavage was repeated every 10 min until the P/F ratio decreased to less than 100 mmHg and remained stable for 30 min (T_ARDS_); then, FiO_2_ was set at 0.4.

**Fig. 1:**
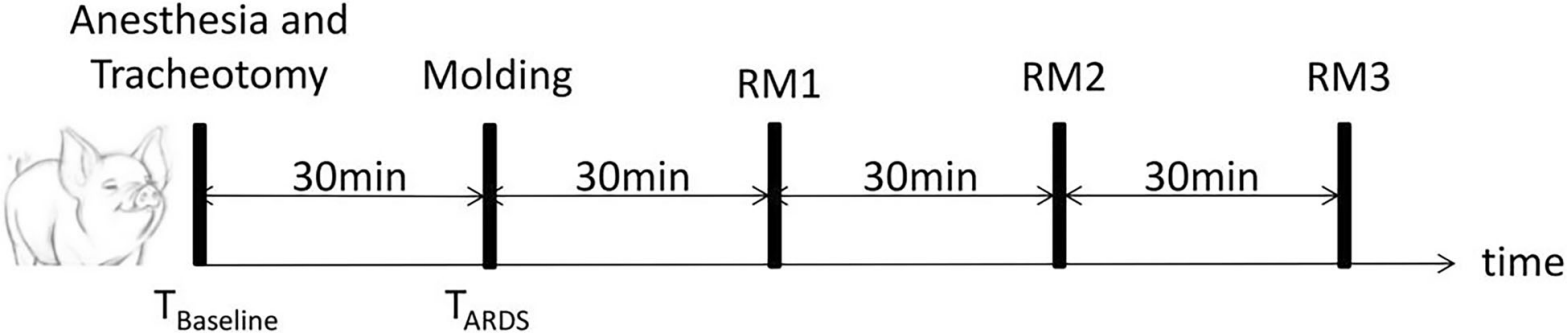
Flowchart of study design. ARDS pigs underwent three sequential recruitment maneuvers applied in random order according to a random number table, with 30 mins at a PEEP of 5 cmH_2_O between maneuvers. Respiratory mechanics, hemodynamic parameters, arterial blood gas and EIT were recorded at T_Baseline_, T_ARDS_, and before and after each recruitment maneuver

ARDS pigs underwent three sequential recruitment maneuvers, including sustained inflation, increments of PEEP and PCV applied in random order according to a random number table, with 30 mins at a PEEP of 5 cmH_2_O between maneuvers (Fig. 1). Circulatory and lung mechanics recovered in 30 min after recruitment maneuvers [13] and a PEEP of 5 cmH_2_O represented physiologic PEEP. Sustained inflation was performed using continuous positive airway pressure (CPAP) held at 40 cmH_2_O for 40 s [14]. For increments of PEEP, PEEP was increased from 5 cmH_2_O to a maximum of 40 cmH_2_O in 5 cmH_2_O increments, with each increment lasting 30 s, and retuned to 5 cmH_2_O in the reverse process. For PCV, peak pressure was 40 cmH_2_O, inspiratory to expiratory ratio was 1:2, and PEEP was 20 cmH_2_O for 2 min. For increments of PEEP and PCV, respiratory rate was set to 30 breaths/min. Respiratory mechanics, hemodynamic parameters, arterial blood gas, and EIT were recorded at T_Baseline_, T_ARDS_, and before and after each recruitment maneuver. MAP, CVP, and PAWP were monitored using calibrated pressure transducers. Blood gases were evaluated with an automated blood gas analyser (Nova M; Nova Biomedical, Waltham, MA, USA).

### EIT measurements and analysis

EIT measurements (PulmoVista 500; Dräger Medical GmbH, Lübeck, Germany) were performed for 3 min each at T_Baseline_, T_ARDS_, and before and after each recruitment maneuver as previously described [15]. EIT data were generated by applying small alternate electrical currents through 16 electrodes located equidistant apart on a belt positioned around the pigs’ thorax, 5 cm above the xyphoid process. A reference electrocardiogram (ECG) electrode was positioned on the abdomen. Current applications and voltage measurements were automatically selected to be compatible with the image reconstruction algorithm. The images were continuously recorded and reconstructed at 40 Hz (Draeger EIT Data Analysis Tool 6.1).

Four regions of interests (ROI) of the same size and shape consisting of contiguous pixels were identified within EIT images obtained during tidal breathing. A cross section of the lung (ventral to dorsal) was divided into four equal parts, namely ROI1, ROI2, ROI3 and ROI4. The upper quarter corresponded to ROI1 and the second quarter from the top to the bottom corresponded to ROI2. The third quarter and the fourth quarter respectively corresponded to ROI3 and ROI4. ROI1 and 2 correspond to non-dependent regions and ROI3 and 4 correspond to gravity-dependent regions (Additional file 1) [16]. For each breathing cycle, the impedance change of the lung was calculated as the impedance difference between end-inspiration and end-expiration of the transverse section image. ∆Z_ROI_ was defined as the impedance change of an ROI [5]. EIT-estimated regional compliance was calculated as ∆Z_ROI_/driving pressure [17]. Tidal volume distribution within the lung was quantified using the global inhomogeneity index, as previously described [18], and analysis of the global inhomogeneity index was performed using customized software developed by Zhao (evaluation_perfusion.exe). For each breathing cycle, the median value of a tidal image, in which each pixel represented the difference in impedance between end-inspiration and end-expiration, was calculated. The absolute difference between the median value and every pixel value was summed to indicate the variation in the Vt distribution. The global inhomogeneity index was adjusted by normalization to the sum of the impedance values. A smaller global inhomogeneity index represented a more homogeneous distribution, and a larger global inhomogeneity index indicated a more inhomogeneous ventilation. The decrease in global inhomogeneity index (ΔGI) with each recruitment maneuver was calculated as the difference in global inhomogeneity index before and after recruitment.

General anesthesia was maintained throughout the study. After completion of the experiments, the animals were in deep anesthesia with propofol (2 mg/kg·h), fentanyl citrate (1 μg/kg·h), midazolam (0.1 mg/kg·h), and atracurium (0.4 mg/kg·h). They were euthanized by a bolus injection of thiopental (0.1 g/kg) intravenously.

### Statistical analyses

Statistical analyses were performed using SPSS v20 (Chicago, IL, USA). Differences in global inhomogeneity and changes in global and regional end-expiratory lung impedance among different recruitment maneuvers were investigated. Comparisons were made between values obtained before and after each recruitment maneuver. For non-normally distributed data, results are expressed as median and interquartile range, and comparisons were made with the Wilcoxon rank test. For data that was normally distributed, results are expressed as mean and standard deviation, and comparisons were made with paired samples t tests and Bonferroni correction. *p* < 0.05 was considered statistically significant.

## Results

In all ten pigs, ARDS was successfully induced with a mean 2.8 ± 1.03 L (2800 ± 1032.80 ml) bronchoalveolar lavages. Mean P/F was significantly decreased after the final lavage (81.69 ± 55.79 mmHg) compared to baseline (362.48 ± 117.38 mmHg).

There were no significant differences in hemodynamic parameters after recruitment with the different maneuvers (Table 1). No animals died during the experiments.

**Table 1:** Hemodynamic and oxygenation parameters before and after recruitment maneuvers

	SI			IP			PCV		
	Before	After	*p*	Before	After	*p*	Before	After	*p*
HR (BPM)	89.1 ± 25.32	97.5 ± 31.17	0.517	90.4 ± 39.40	96.9 ± 46.84	0.950	93.8 ± 38.44	94.1 ± 41.04	0.987
MAP (mmHg)	102.1 ± 23.14	92.7 ± 17.71	0.321	109.2 ± 19.00	96.8 ± 23.93	0.455	109.1 ± 20.26	96.6 ± 23.53	0.219
CVP (mmHg)	7.62 ± 3.37	8.81 ± 3.12	0.420	7.45 ± 2.91	9.10 ± 4.72	0.523	7.42 ± 2.67	9.46 ± 3.41	0.161
PAWP (mmHg)	8.81 ± 4.94	10.72 ± 4.40	0.376	9.34 ± 4.08	11.62 ± 4.88	0.349	9.17 ± 3.67	10.83 ± 4.59	0.372
CO (L/min)	4.74 ± 1.55	4.45 ± 1.35	0.664	4.74 ± 2.11	4.46 ± 1.63	0.733	4.53 ± 1.67	4.48 ± 1.63	0.945
pH	7.28 ± 0.12	7.29 ± 0.12	0.95	7.27 ± 0.13	7.30 ± 0.12	0.627	7.27 ± 0.12	7.31 ± 0.12	0.468
PaCO_2_ (mmHg)	52.56 ± 13.82	48.24 ± 13.20	0.484	55.82 ± 17.49	45.94 ± 13.82	0.206	56.2 ± 16.15	46.09 ± 13.70	0.568
PaO_2_ (mmHg)	81.62 ± 22.36	145.83 ± 26.86^a^	0.000	78.22 ± 24.28	167.98 ± 36.85^a^	0.000	77.54 ± 24.69	155.83 ± 50.85^a^	0.000
SaO_2_	86.77 ± 8.28	96.46 ± 2.05^a^	0.002	84.91 ± 8.25	97.57 ± 1.96^a^	0.000	84.63 ± 8.08	94.93 ± 6.52^a^	0.006
P/F (mmHg)	81.62 ± 22.36	145.83 ± 26.86^a^	0.000	78.22 ± 24.28	167.98 ± 36.85^a^	0.000	77.54 ± 24.69	155.83 ± 50.85^a^	0.000
Cr (ml/cmH_2_O)	13.34 ± 3.66	24.26 ± 8.00^a^	0.001	12.88 ± 3.20	27.51 ± 7.99^a^	0.000	13.01 ± 3.09	26.67 ± 8.60^a^	0.000
HCO_3_^−^(mmol/L)	24.13 ± 2.99	23.02 ± 3.25	0.437	24.8 ± 3.73	22.08 ± 3.79	0.144	25.01 ± 3.33	22.54 ± 3.90	0.148

*HR* heart rate; *MAP* mean arterial pressure; *CVP* central venous pressure *PAWP* pulmonary artery wedge pressure; *CO* cardiac output; *PaCO*_*2*_ partial pressure of arterial carbon dioxide; *PaO*_*2*_ partial pressure of arterial oxygen; *SaO*_*2*_ arterial oxygen saturation; *P/F* ratio of partial pressure of arterial oxygen to fraction of inspired oxygen; *Cr* respiratory system compliance; *SI* sustained inflation; *IP* increments of PEEP; *PCV* pressure-controlled ventilation;

^a^ < 0.05 versus Before

PaO_2_, arterial oxygen saturation, and P/F were significantly improved after recruitment with sustained inflation, increments of PEEP or PCV (all *p* < 0.05), and there were no significant differences between maneuvers. The recruitment maneuvers had no effect on PaCO_2_ or pH (Table 1).

**Fig. 2:**
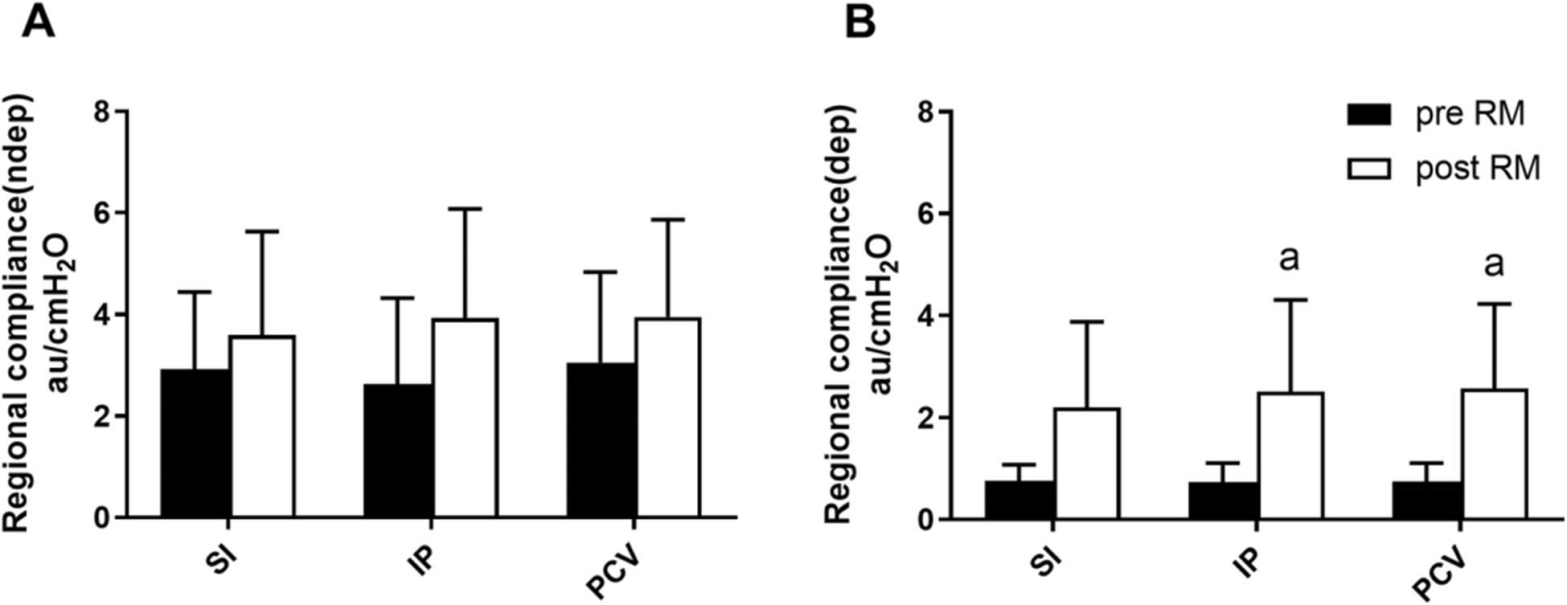
Compliance in non-gravity-dependent (**a**) and gravity-dependent (**b**) lung regions. Compliance in different regions was calculated by dividing ∆Z_ROI_ by tracheal pressure changes assuming no flow at the end of inspiration and expiration. Compliance = ∆Z_ROI_/driving pressure. ^a^*p* < 0.05, comparison between before and after recruitment maneuver. SI, sustained inflation; IP, increments of PEEP; PCV, pressure-controlled ventilation; RM, recruitment maneuvers; ROI, regions of interest; ∆ Z_ROI,_ the regional impedance change for a ROI

Overall respiratory system compliance was significantly increased after recruitment with sustained inflation, increments of PEEP, or PCV (p < 0.05) (Table 1). The recruitment maneuvers had no significant effect on compliance in non-gravity-dependent lung regions. Compliance was significantly increased in gravity-dependent lung regions after lung recruitment with increments of PEEP or PCV (preIP 0.74 ± 0.37 au/cmH_2_O vs. postIP 2.51 ± 1.80 au/cmH_2_O, *p* = 0.025; prePCV 0.75 ± 0.36 au/cmH_2_O vs. postPCV 2.78 ± 1.65 au/cmH_2_O, *p* = 0.012), but there were no significant differences in compliance between maneuvers (Fig. 2).

**Fig. 3:**
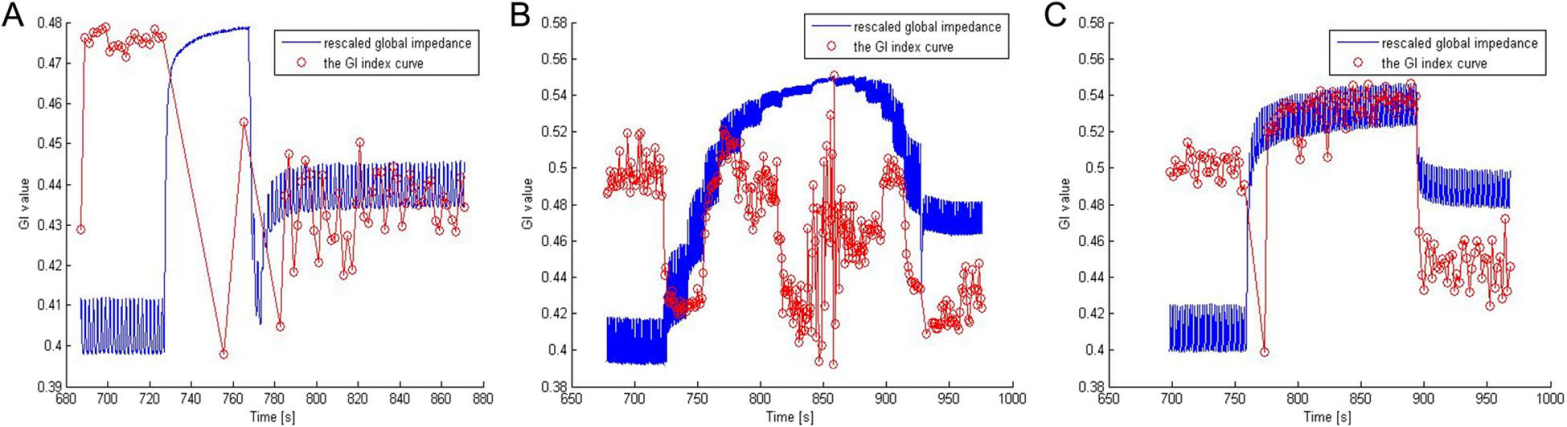
EIT-based global inhomogeneity index with recruitment A: SI; B: IP; C: PCV. Recruitment maneuvers were performed in the same pig. The figure was exported by a data analysis tool, and the scales cannot be adjusted. Blue lines indicate rescaled global impedance, and red circles indicate the global inhomogeneity index. The global inhomogeneity index increased during recruitment with PCV, and varied during recruitment with SI and IP. The global inhomogeneity index was significantly decreased after recruitment with SI, IP, or PCV. SI, sustained inflation; IP, increments of PEEP; PCV, pressure-controlled ventilation; GI, global inhomogeneity

**Fig. 4:**
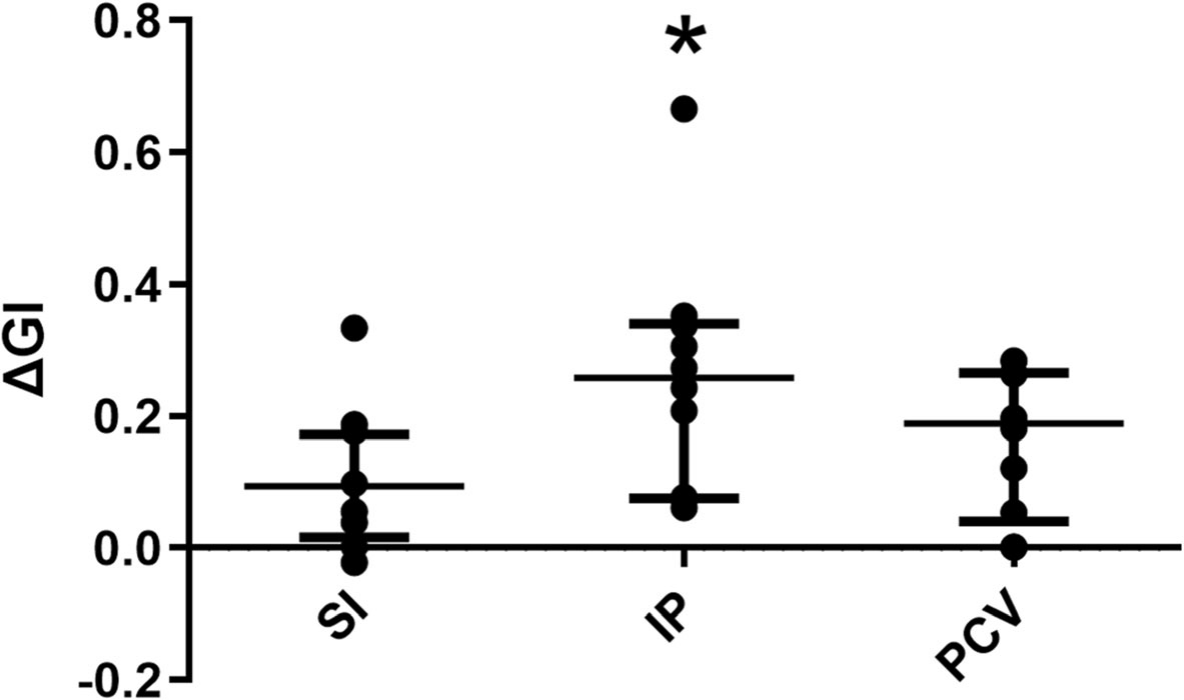
Decrease in EIT-based global inhomogeneity index (∆GI) after recruitment. *p < 0.05 SI vs. IP

Global inhomogeneity index was significantly decreased after recruitment with sustained inflation, increments of PEEP, or PCV (preSI 0.55 ± 0.14u vs. postSI 0.42 ± 0.040; preIP 0.62 ± 0.19u vs. postIP 0.42 ± 0.07u; prePCV 0.60 ± 0.09u vs. postPCV 0.4431 ± 0.05u; all *p* < 0.001) (Fig. 3). The ΔGI was significantly greater after recruitment with increments of PEEP compared to sustained inflation (*p* = 0.023), but there was no difference in ΔGI between increments of PEEP and PCV or between sustained inflation and PCV (Fig. 4).

## Discussion

This study used EIT to investigate the physiological effects of different recruitment maneuvers that achieve the same maximum pressure when held for different time spans, including sustained inflation, increments of PEEP and PCV, for alveolar recruitment in a pig model of ARDS. Findings showed that these recruitment maneuvers increased oxygenation and compliance in overall and gravity-dependent lung regions, and decreased inhomogeneous gas distribution in the ARDS lung, with no adverse effects on hemodynamics immediately after the maneuver. In a previous study [19], recruitment maneuvers transiently but profoundly depressed cardiac output in three models of acute lung injury. These results imply that a lung recruiting maneuver should be applied with caution, especially when using sustained inflation in the setting of pneumonia.

Patients with ARDS can suffer from inhomogeneous gas distribution, which leads to ventilation–perfusion mismatching, a high dead-space fraction, and the potential for ventilator-induced lung injury (VILI). Recruitment maneuvers aim to open collapsed alveoli and improve oxygenation and respiratory system compliance. However, recruitment maneuvers can over-distend aerated alveoli, and ventilation at high inflation pressures can lead to VILI.

Heterogeneous lung structure (i.e, collapsed and overexpanded contiguous lung regions) is increasingly recognized as a key risk factor for inhomogeneous gas distribution, VILI, and mortality in mechanically ventilated patients [20]. Recent studies showed that the extent of lung inhomogeneities increase with the severity of ARDS [21], and a protective ventilatory strategy may not be sufficient to minimize VILI in patients with ARDS whose disease process is characterized by an inhomogeneous distribution of pulmonary lesions that includes a small, nondependent, normally aerated compartment and a large, dependent, nonaerated compartment [22, 23].

In the present study, the inhomogeneous distribution of lung alterations in the pig model of ARDS was directly assessed using EIT. EIT has several advantages compared to established imaging techniques such as computed tomography as it is radiation free and applicable at the bedside; however, computation of the global inhomogeneity index is not a bedside technique as it requires offline measurements. In previous studies, Zhao [17] et al. developed the global inhomogeneity index to quantify the spatial extent and dispersion in the distribution of tidal breath, reporting that a larger global inhomogeneity index reflected more inhomogeneity between lung units. A tidal EIT image is generated and variations in pixel values are used as an indicator of the inhomogeneity of air distribution during tidal ventilation [17]. In the present study, we used the global inhomogeneity index as a direct representation of global inhomogeneity in tidal ventilation in ARDS pigs. As the global inhomogeneity index is 0.40 ± 0.05 in patients under anesthesia without pulmonary disease [17], the global inhomogeneity index was expected to be > 0.45 in our experimental animals. We assessed the change in inhomogeneity with various recruitment maneuvers. Previous studies have shown different recruitment maneuvers are associated with differences in oxygenation, respiratory system compliance, hyperinflation, and hemodynamics [13, 24–26]. However, a ventilation strategy with aggressive lung recruitment may increase mortality in patients with ARDS [27]. The present study showed that increments of PEEP significantly improved inhomogeneity of the lung compared to sustained inflation in ARDS pigs, while there was no difference between increments of PEEP and PCV or between sustained inflation and PCV. These data suggest that evaluating the effect of recruitment maneuvers with EIT could play a role in minimizing VILI. Results of this study should be extrapolated to the clinical setting with caution, considering the differences in the shape of the thorax between pigs and humans. Clinical trials are required to evaluate the efficacy and safety of recruitment maneuvers in patients with ARDS, and current evidence does not support the use of recruitment maneuvers in clinical practice.

Our study was associated with several limitations. First, we measured hemodynamic parameters after not during recruitment maneuvers. A previous study [19] recorded hemodynamic parameters during and after recruitment maneuvers. Cardiac output was transiently decreased during recruitment maneuvers, there were no sustained hemodynamic effects following recruitment maneuvers, and no difference was found among recruitment maneuvers, which was consistent with our research. Second, the relative impedance changes monitored by EIT may have been affected by cardiac movement. Errors in the reconstruction algorithm and resorption atelectasis could not be measured as EIT was used for monitoring dynamic ventilation distribution. Third, the decrease in global inhomogeneity index in the different ROIs would be very informative. Unfortunately, our analytical software can only generate a global value. Last, maximal recruitment of the lung was not achieved with any maneuver. Failure to achieve maximal recruitment of the lung would affect monitoring of end-expiratory lung impedance. A peek pressure of 40 cmH_2_O may not have been sufficient for opening certain alveoli in ARDS pigs. Borges [28] et al. reported that when PEEP was set to 25 cm H_2_O in patients with ARDS, producing peak airway pressures of 40 cm H_2_O, lung recruitment was approximately 67%. When peak airway pressures of 60 cm H_2_O were reached, lung recruitment was approximately 87%. Maximal recruitment would further improve the heterogeneity of the lung, but with a concrete risk of damaging the nondependent normally aerated compartments.

## Conclusions

This study used EIT to show that different recruitment maneuvers that achieve the same maximum pressure, including sustained inflation, increments of PEEP, and PCV, increased oxygenation and overall and EIT- estimated regional compliance, and decreased inhomogeneous gas distribution. Increments of PEEP significantly improved inhomogeneity of the lung compared to sustained inflation and PCV. Further studies are needed to confirm the clinical significance of these findings.

## Supplementary information

**Additional file 1.** Schematic diagram of EIT image partitioning.

## Data Availability

The datasets used during the current study are available from the corresponding author on reasonable request.

## Abbreviations

ARDS: Acute respiratory distress syndrome
EIT: Electrical impedance tomography
PaO2: Arterial partial pressure of oxygen
FiO2: Fraction of inspired oxygen
P/F ratio: Arterial partial pressure of oxygen /fraction of inspired oxygen
PEEP: Positive end-expiratory pressure
PCV: Pressure-controlled ventilation
ΔGI: Decrease in global inhomogeneity index
MAP: Mean arterial pressure
Vt: Tidal volume
I:E: Inspiration-to-expiration time ratio
CVP: Central venous pressure
PAWP: Pulmonary arterial wedge pressure
PiCCO: Pulse contour cardiac output
T_Baseline_: Time of baseline
T_ARDS_: Time of the ARDS model remained stable for 30 min
CPAP: Continuous positive airway pressure
ECG: Electrocardiogram
ROI: Regions of interests
VILI: Ventilator-induced lung injury
