# Supplementary figures and images for: Physiological effects of different recruitment maneuvers in a pig model of ARDS
Source: BMC Anesthesiol. 2020 Oct 21;20:266. doi: 10.1186/s12871-020-01164-x (PMC7576861; doi:10.1186/s12871-020-01164-x)

## Schematic diagram of EIT image partitioning

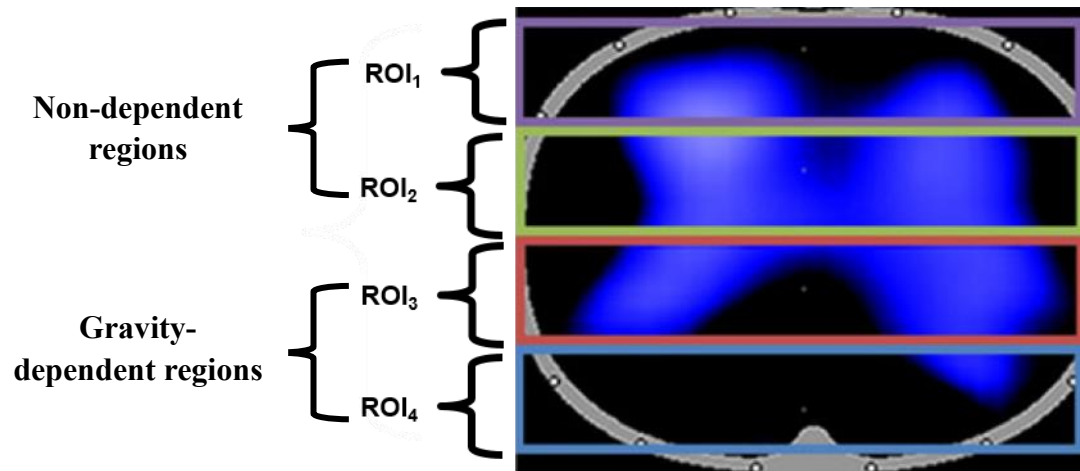

Supplement: Supplementary file 1 — Additional file 1. Schematic diagram of EIT image partitioning. [file 12871_2020_1164_MOESM1_ESM.pdf]
